# Low autonomic arousal as a risk factor for reoffending: A population-based study

**DOI:** 10.1371/journal.pone.0256250

**Published:** 2021-08-20

**Authors:** Sofi Oskarsson, Ralf Kuja-Halkola, Antti Latvala, Anneli Andersson, Miguel Garcia-Argibay, Bridget M. Bertoldi, Adrian Raine, Christopher J. Patrick, Henrik Larsson, Catherine Tuvblad

**Affiliations:** 1 School of Psychology, Law and Social Work, Örebro University, Örebro, Sweden; 2 Department of Medical Epidemiology and Biostatistics, Karolinska Institutet, Solna, Sweden; 3 Institute of Criminology and Legal Policy, University of Helsinki, Helsinki, Finland; 4 School of Medical Sciences, Örebro University, Örebro, Sweden; 5 Department of Psychology, Florida State University, Tallahassee, Florida, United States of America; 6 Department of Criminology, Psychiatry, and Psychology, University of Pennsylvania, Philadelphia, Pennsylvania, United States of America; 7 Department of Psychology, University of Southern California, Los Angeles, California, United States of America; University of Jyvaskyla, FINLAND

## Abstract

**Background:**

Low resting heart rate (RHR) and low systolic blood pressure (SBP) are associated with criminal behavior. However, knowledge is lacking about their predictive value for reoffending.

**Aim:**

We aimed to examine associations of RHR and SBP with reoffending in a large population-based sample.

**Methods:**

We conducted a cohort study of all convicted male conscripts born in Sweden 1958–1990 (N = 407,533). We obtained data by linking Swedish population-based registers. Predictor variables were RHR and SBP, measured at conscription which was mandatory until 2010 for men at age 18. The outcome variable was reoffending, defined as criminal convictions (any crime, violent crime and non-violent crime), obtained from the National Crime Register. We used survival analyses to test for associations of RHR and SBP with reoffending, adjusting for pertinent covariates such as socioeconomic status, height, weight and physical energy capacity.

**Results:**

In fully adjusted Cox regression models, men with lower RHR (≤60 bpm) had higher risk of reoffending (any crime: HR = 1.17, 95% CI: 1.14, 1.19, violent crime: HR = 1.23, 95% CI: 1.17, 1.29, non-violent crime: HR = 1.16, 95% CI: 1.14, 1.19), compared to men with higher RHR (≥ 82 bpm). Men with lower SBP (≤80 mmHg) had higher risk of reoffending (any crime: HR = 1.19, 95% CI: 1.17, 1.21, violent crime: HR = 1.16, 95% CI: 1.12, 1.20, non-violent crime: HR = 1.20, 95% CI: 1.18, 1.22), compared to men with higher SBP (≥138 mmHg).

**Conclusions:**

Low autonomic arousal is associated with increased risk of reoffending. RHR and SBP should be investigated further as potential predictors for reoffending as they each may have predictive value in risk assessment protocols.

## Introduction

Reoffending (i.e., being convicted of a criminal offense more than once) poses a significant burden on society and constitutes a serious public health problem [1]. As such, it is important to identify individuals with the highest risk of reoffending to better target interventions. Although there are over 300 risk assessment protocols incorporating known risk factors for reoffending (e.g., history of criminal behavior, employment) [2], they show low to moderate positive predictive value and high negative predictive value [3]. Thus, current risk assessment tools are likely to efficiently identify individuals with low risk of reoffending, but they may in parallel result in a vast number of false positive decisions, as they tend to predict who will reoffend with only moderate precision. Therefore, it is important to consider other risk factors for reoffending that can add predictive value.

Previous research has contributed to the identification of important predictors for criminal behavior by examining the role of the autonomic nervous system [4]. Studies using large scale population-based data have shown that *low* resting heart rate (RHR) increases the risk for criminal behavior [5, 6], an association also confirmed in a meta-analysis [7]. The same meta-analysis emphasized the importance of continuing to incorporate low RHR into research on criminal behavior, to confirm low RHR as a correlate of this outcome [7]. In contrast, *high* RHR relates to psychiatric disorders involving anxious and depressive symptoms [6]. As such, high RHR is related to a range of psychiatric disorders, while low RHR is specifically related to externalizing behavior problems including criminal behavior [6, 8]. A recent family-based study extended these findings by presenting evidence that the association between RHR and criminal behavior was largely due to familial confounding (i.e., genetic and environmental factors influencing both pulse and crime), whereas RHR directly increased the risk for anxiety/depression [9].

While the body of research linking low RHR to criminal behavior is impressive, little is known about its relationship with reoffending. Only one previous study has tested for a relationship between RHR and reoffending, using a sample of 68 delinquent adolescent men [10]. The results showed no association between low RHR and reoffending (according to police registers), but attenuated heart rate reactivity and increased heart rate variability with a stressor task did predict a higher likelihood of reoffending over a 5-year follow-up period. Research using larger and more diverse samples is needed to further evaluate the relationship between RHR and reoffending.

In addition to RHR, systolic blood pressure (SBP) also reflects autonomic arousal [11]. RHR and SBP are each influenced by both the sympathetic and the parasympathetic branches of the nervous system, with the parasympathetic branch being dominant in resting conditions [11]. Despite their shared physiological basis, RHR and SBP are two separate measures and do not always rise and fall in parallel. Further, the body of research linking low RHR to criminal behavior is continuously expanding, yet SBP has received little attention by comparison. Work thus far has reported associations for low SBP with prior convictions in an institutionalized sample [12], and to criminal convictions in two large scale population-based studies [5, 6]. Similar to elevated RHR, prior work has shown higher SBP to be associated with an increased risk of psychiatric disorders (e.g., obsessive-compulsive disorder) [6]. Low SBP has also been linked to aggression in adolescent boys [13] and to relational aggression in older pre-school children [14], but no study has yet tested for an association between SBP and reoffending.

The mechanisms underlying the association between autonomic arousal and criminal behavior are not yet known, but two theories have dominated the literature thus far. Fearlessness theory suggests that a “resting state” is in reality a mildly stressful experience, as participants are assessed within a novel lab setting [4]. The lab environment can induce the “white coat effect”, which refers to the increased heart rate and blood pressure that some individuals experience while undergoing a clinical assessment [15]. As such, low RHR in this situation may reflect a lack of anticipatory fear that relates in turn to an increased risk for criminal behavior–since such acts typically involve elements of personal risk and adverse consequences. From this perspective, fearless individuals may lack the negative emotional response that typically inhibits the general population from engagement in antisocial behaviors in the first place. Individuals who do not fear the consequences of their behavior, such as the possibility of punishment for a crime, could be expected to exhibit lower autonomic arousal in unfamiliar situations [4].

Another theoretical explanation for the association between autonomic arousal and criminal behavior is the stimulation-seeking theory, which conceives of low autonomic arousal as an unpleasant physiological state, that can prompt individuals to engage in activities that elevate their arousal to more optimal levels [4, 16]. According to this perspective, criminal behavior is one form of activity through which under-aroused individuals can relieve physiological discomfort. Similar to fearlessness theory, individuals with low autonomic arousal are expected to be at greater risk for reoffending given their need to seek stimulation to alter their physiological state. From these theoretical perspectives, individuals showing low autonomic arousal within a lab-test situation are predicted to be more likely to reoffend.

To extend existing knowledge regarding the potential predictive validity of low autonomic arousal for reoffending, we examined associations for both RHR and SBP with reoffending in a large-scale population-based sample of men in Sweden with criminal conviction histories. We specifically sought to address whether 1) lower RHR in previously convicted men is associated with an increased risk of reoffending, and 2) whether lower SBP in previously convicted men is also associated with an increased risk of reoffending.

## Material and methods

### Data source

We linked several Swedish nation-wide population-based registers via the unique personal identity numbers that has been assigned since 1947 to all Swedish citizens at birth or immigration [17]. The PIN enables unambiguous linkage across several national registers with data that is collected by professional and administrative personnel. The record linkage was approved by the Regional Ethical Review Board in Stockholm (2013/862-31/5). Linkage between the registers was based on anonymized personal identity numbers. We used the following registers: (i) The Total Population Register (TPR) was established in 1968 and includes all individuals with a permanent residency in Sweden. TPR contains demographic data such as sex, age, death and migration [18], (ii) The Swedish Military Conscription Register was established to register military recruits and contains information from a 2-day conscription assessment, mandatory until 2010 for men at age 18 in Sweden [19]. This assessment was aimed at evaluating each individual’s ability to carry out military service and suitability for different positions within the military service [20]. Failure to report for conscription without excusable grounds (i.e., medical illness and/or mental illness) was a punishable offense up to the year 2010. As such, 95% of all men in Sweden were conscripted around age 18 during the 1990’s [20], (iii) The National Crime Register contains data on all individuals with a criminal conviction from a Swedish district court at or after age 15, the minimum age of criminal responsibility in Sweden, (iv) National Census records which contains information such as socioeconomic status.

### Study population

A total of 1,508,974 men born in Sweden between 1958 and 1990 were identified from the TPR. We excluded those without a conscription date (n = 33,499), and those who participated in the conscription assessment before age 17 or after age 19 (n = 14,520) to minimize potential effects of age and developmental changes. The mean age at conscription in the present sample was 18.2 years (*standard deviation (SD) = 0*.*4*). We further excluded men without at least one conviction (n = 969,366). The mean age at first conviction in the present sample was 21.6 years (*SD = 7*.*4*, *median (Md) = 19*.*0*, *1*^*st*^ *quartile (q1) = 16*.*0*, *q3 = 24*.*0)*. The mean age for reoffending was 24.7 (*SD* = 8.3, *Md* = 21.8, *q1* = 18.5, *q3* = 28.5), and the mean time until first reoffending was 4.9 years (*SD* = 6.0). Also excluded were men with an immigration status at any point in time, or an emigration status before conscription (n = 23,537), as determined from the Swedish Migration Register, which comprises part of the TPR. Also, if the first conviction happened after conscription, we excluded men who emigrated or died before the date of conviction (n = 179). Lastly, we excluded those who had missing values on both RHR and SBP (n = 60,340). The final analysis sample thus included 407,533 men.

### Predictors

#### Resting heart rate and systolic blood pressure

As part of the enlistment protocol, conscripts underwent a physical assessment in which RHR and SBP were recorded using a pneumatic arm-cuff monitor positioned at heart level. Measurements were taken from conscripts while laying down in the supine position, following 5 to 10 minutes of rest. This procedure is the most common approach to obtaining measures of RHR and SBP in clinical settings in Sweden and may therefore reflect how these measures are obtained in primary care clinics. More detailed information has been provided elsewhere [21]. In line with previous work, individuals with a RHR below 35 beats per minute (bpm) and above 145 bpm were excluded as outliers [5, 22]. A valid value for RHR was available for almost two thirds of the sample (n = 230,141, 56.5%). Reasons for the missing RHR data have been reported elsewhere [5, 21]. Additionally, individuals with SBP values below 80 millimeter of mercury (mmHg) and above 160 mmHg were excluded as outliers [5], resulting in 406,801 men (99.8%) with valid values for SBP.

### Outcome measure

#### Reoffending

In our primary analyses, reoffending served as the outcome variable and was defined as a conviction for any type of crime among men with any type of previous conviction. We further divided the reoffense-outcome (i.e., criminal convictions) into convictions for violent versus non-violent crimes. In line with previous research, violent crimes included homicide, manslaughter, assault, kidnapping, illegal confinement, unlawful coercion, gross violation of a person’s or a woman’s integrity, unlawful threats, intimidation, robbery, arson, and threats and violence against an officer [23]. Crimes other than these were considered non-violent [24].

### Covariates

From the Swedish Military Conscription Register, we included height and weight as covariates in the analyses because of their potential associations with autonomic arousal and reoffending [25]. We also covaried for physical energy capacity (in Watts), assessed during conscription using a cycle ergometer [26], and adjusted by weight to account for body size [5], as this is a well-known correlate of autonomic arousal [27], and potentially of reoffending [5]. In addition, we covaried for childhood socioeconomic status (SES), a well-established risk factor for criminal behavior [28], coded from National Census records as low, medium or high based on the occupation listed for the head of each participant’s household [5]. Lastly, we adjusted for birth year to account for potential cohort effects and age at first criminal conviction.

### Statistical analysis

Data management and analyses were performed using version 9.4 of the SAS software package (SAS Institute Inc., Cary, NC) and R 3.6.1 [29]. We used survival analyses, time-to-event, to test for associations of RHR and SBP as predictor variables with reoffending as the outcome variable. Cox proportional hazard models were used to estimate both crude and adjusted relative hazards of reoffending during follow-up, together with 95% confidence intervals (CIs). We conducted one Cox proportional hazards regression analysis for each reoffense-outcome (i.e., conviction for any type of crime, violent crime, and non-violent crime). A hazard ratio (HR) greater than 1 indicate an increased risk of the outcome, and a HR below 1 indicate a reduced risk of the outcome. The large sample size in the present study allowed for detection of any violation of the proportionality assumption. The proportional hazards assumption was verified by visually inspecting the Kaplan Meier curves (S1 and S2 Figs).

In our main analyses, the follow-up began from the date that participants were first convicted of their first crime of any kind (i.e., violent or non-violent), whether before or after conscription, and continued until they either 1) experienced the reoffense-outcome of analytic interest (i.e., conviction for any type of crime, for a violent crime or for a non-violent crime), 2) emigrated, 3) died, or 4) reached the end of the study period (31 December 2013).

RHR, SBP, weight, height and physical capacity were divided into quintiles to allow for potential non-linear relationships. Birth year was divided into approximately equal categories (1957–1964, 1965–1969, 1970–1974, 1975–1979, 1980–1984, 1985–1990), and age at first crime was categorized in terms of years (15, 16, 17, 18, 19, 20, 21–25, 26–30, 31–35, 36–40, 41–45, 46–50, 51–55).

Three models were conducted for each reoffense-outcome. The first model did not adjust for any of the covariates. The second model adjusted for birth year, age at first crime and childhood SES. In the third model, height, weight, and physical energy capacity were included as additional covariates. All models accounted for the non-independence of siblings (brothers) within the sample by using a cluster-robust sandwich estimator.

### Sensitivity analyses

To rule out potential alternative explanations for observed associations and test the robustness of our estimates, we conducted a series of sensitivity analyses. To explore the extent to which our estimates were affected by the reoffense-outcome occurring prior to, or after conscription, we stratified the sample based on whether the first conviction had occurred before (n = 157,517) or after (n = 250,016) conscription. We followed the first sub-sample from the day of conscription until the reoffense-outcome. The second sub-sample was followed from the day of their first conviction until the reoffense-outcome. For both sub-samples, we conducted separate fully adjusted models for each reoffense-outcome: any type of crime, violent crime, and non-violent crime.

To evaluate whether estimates regarding non-violent crime were driven by individuals having convictions for both violent and non-violent crime, we conducted a sensitivity analysis that included only men with no violent convictions at any point in time.

All analyses were conducted using time since last event (date of criminal conviction or date of conscription) as the underlying temporal metric. To make sure that our estimates were not affected by this approach, all analyses were re-conducted using age as the underlying temporal metric. We also conducted a sensitivity analysis using the date of the crime, rather than date of conviction, when applicable. Lastly, we ran analyses with a restricted sample including only men with valid RHR and SBP data (n = 229,409). All sensitivity analyses are presented in the S1–S12 Tables.

## Results

Baseline characteristics of the full sample are shown in Table 1. The Kaplan-Meier survival curves for RHR and SBP are shown in the S1 and S2 Figs.

**Table 1:** Descriptive information for convicted men.

Variable	No. (%) with data	M (SD)
Age at conscription, y	407,533 (100.0)	18.2 (0.4)
Age at first conviction, y	407,533 (100.0)	21.6 (7.4)
Age at first violent conviction, y	79,723 (19.6)	24.9 (8.2)
Age at first non-violent conviction, y	392,630 (96.3)	22.1 (7.4)
Age at reoffending, y	191,359 (47.0)	24.7 (8.3)
Age at violent reoffending, y	25,446 (6.2)	27.4 (8.4)
Age at non-violent reoffending, y	178,120 (43.7)	24.8 (8.3)
Resting heart rate, bpm	230,141 (56.5)	71.9 (12.5)
Systolic blood pressure, mmHg	406,801 (99.8)	127.8 (10.6)
Weight, kg	406,500 (99.8)	70.9 (11.2)
Height, cm	406,567 (99.8)	179.0 (6.5)
Physical capacity, W	371,313 (91.1)	276.6 (50.4)
**SES**		
Low	174,772 (42.9)	
Medium	130,437 (32.0)	
High	66,761 (16.4)	
Missing	35,563 (8.7)	

Abbreviations: y (years), bpm (beats per minute), mmHg (millimeter of mercury), kg (kilogram), cm (centimeter), W (Watt).

### Associations between autonomic arousal and reoffending

**Fig 1:**
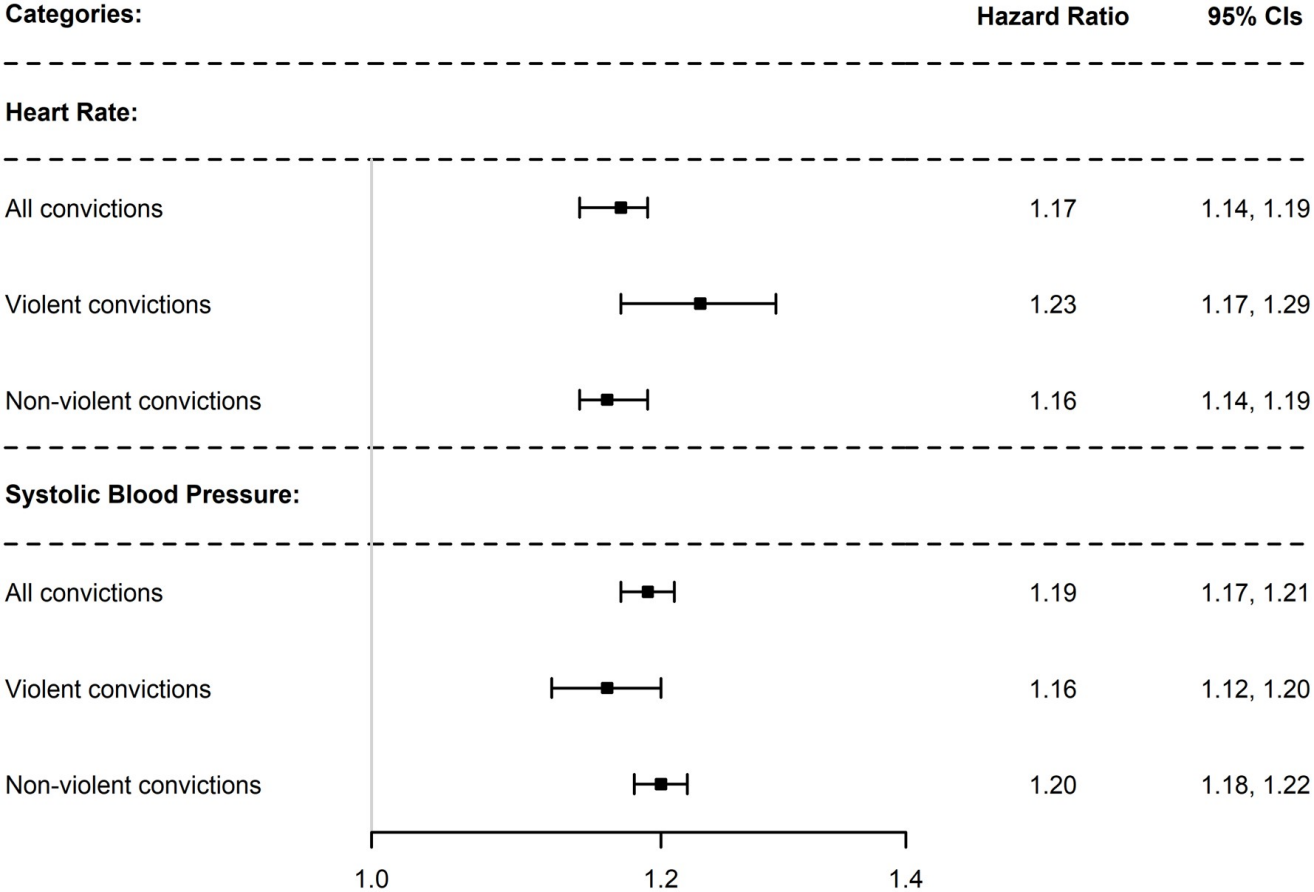
Forest plot displaying hazard ratios and 95% confidence intervals for the associations between resting heart rate and systolic blood pressure with reoffending. Estimates represents comparison between convicted males in the lowest quintile and convicted males in the highest quintile. Models are adjusted for birth year, age at first crime, SES, height, weight and physical capacity.

Fig 1 displays an overview of the estimates from the main analyses for the fully adjusted models for RHR and SBP. All models showed that lower RHR was associated with a higher risk of reoffending, independent of the type of crime. Adjusting for covariates resulted in higher estimates in each step. The fully adjusted models showed that men in the lowest quintile of RHR (≤ 60 bpm), compared to those in the highest quintile (≥ 82 bpm) had a 17% higher risk of reoffending with a conviction for any type of crime (HR = 1.17, 95% CI: 1.14, 1.19), a 23% higher risk of reoffending with a conviction for a violent crime (HR = 1.23, 95% CI: 1.17, 1.29), and a 16% higher risk of reoffending with a conviction for a non-violent crime (HR = 1.16, 95% CI: 1.14, 1.19; Table 2).

**Table 2:** Cox proportional hazard regression models for resting heart rate with reoffending.

	Hazard Ratio (95% CI)		
Quintiles for RHR in bpm^a^	Unadjusted HRs	Adjusted HRs^a^	Adjusted HRs^b^
**All criminal convictions**			
1^st^ (35–60)	1.06 (1.04, 1.08)	1.08 (1.06, 1.10)	1.17 (1.14, 1.19)
2^nd^ (61–67)	1.07 (1.05, 1.09)	1.07 (1.04, 1.09)	1.12 (1.09, 1.14)
3^rd^ (68–73)	1.07 (1.05, 1.09)	1.06 (1.04, 1.08)	1.10 (1.07, 1.12)
4^th^ (74–81)	1.05 (1.03, 1.07)	1.04 (1.02, 1.07)	1.06 (1.04, 1.08)
5^th^ (82–145)	Reference^c^	Reference	Reference
**Violent convictions**			
1^st^ (35–60)	1.12 (1.07, 1.17)	1.12 (1.07, 1.17)	1.23 (1.17, 1.29)
2^nd^ (61–67)	1.12 (1.07, 1.16)	1.11 (1.06, 1.16)	1.18 (1.13, 1.24)
3^rd^ (68–73)	1.07 (1.02, 1.11)	1.06 (1.01, 1.10)	1.08 (1.03, 1.14)
4^th^ (74–81)	1.04 (0.99, 1.08)	1.02 (0.98, 1.07)	1.04 (0.99, 1.09)
5^th^ (82–145)	Reference	Reference	Reference
**Non-violent convictions**			
1^st^ (35–60)	1.05 (1.03, 1.07)	1.07 (1.05, 1.09)	1.16 (1.14, 1.19)
2^nd^ (61–67)	1.06 (1.04, 1.08)	1.06 (1.04, 1.08)	1.11 (1.09, 1.14)
3^rd^ (68–73)	1.07 (1.05, 1.09)	1.06 (1.04, 1.08)	1.10 (1.07, 1.12)
4^th^ (74–81)	1.05 (1.03, 1.07)	1.04 (1.02, 1.06)	1.06 (1.03, 1.08)
5^th^ (82–145)	Reference	Reference	Reference

Abbreviations: bpm (beats per minute), HR (hazard ratio)

^a^ Adjusted for age at first conviction, birth year, and SES

^b^ Adjusted for age at first conviction, birth year, SES, physical energy capacity, height, and weight

^c^ Category of comparison

In line with the results for RHR and reoffending, all models showed that lower SBP was associated with an increased risk of reoffending, independent of the type of crime. All estimates were attenuated in each model after adjusting for covariates, but there was still evidence of an association in each. The fully adjusted models showed that men in the lowest quintile of SBP (≤ 80 mmHg), compared to those in the highest quintile (≥ 138 mmHg), had a 19% higher risk for reoffending with a conviction for any type of crime (HR = 1.19, 95% CI: 1.17, 1.21), a 16% higher risk for reoffending with a conviction for a violent crime (HR = 1.16, 95% CI: 1.12, 1.20), and a 20% higher risk for reoffending with a conviction for a non-violent crime (HR = 1.20, 95% CI: 1.18, 1.22), Table 3.

**Table 3:** Cox proportional hazard regression models for systolic blood pressure with reoffending.

	Hazard Ratio (95% CI)		
Quintiles for SBP in mmHg	Unadjusted HRs	Adjusted HRs^a^	Adjusted HRs^b^
**All criminal convictions**			
1^st^ (80–119)	1.25 (1.23, 1.26)	1.21 (1.19, 1.22)	1.19 (1.17, 1.21)
2^nd^ (120–123)	1.17 (1.15, 1.19)	1.14 (1.12, 1.16)	1.13 (1.11, 1.15)
3^rd^ (124–129)	1.12 (1.10, 1.14)	1.10 (1.08, 1.11)	1.09 (1.07, 1.11)
4^th^ (130–137)	1.08 (1.07, 1.10)	1.07 (1.05, 1.08)	1.06 (1.05, 1.08)
5^th^ (138–160)	Reference^c^	Reference	Reference
**Violent convictions**			
1^st^ (80–119)	1.19 (1.16, 1.23)	1.14 (1.11, 1.18)	1.16 (1.12, 1.20)
2^nd^ (120–123)	1.12 (1.08, 1.16)	1.08 (1.04, 1.12)	1.09 (1.05, 1.13)
3^rd^ (124–129)	1.07 (1.03, 1.10)	1.06 (1.02, 1.10)	1.06 (1.02, 1.10)
4^th^ (130–137)	1.08 (1.04, 1.11)	1.06 (1.03, 1.10)	1.07 (1.04, 1.11)
5^th^ (138–160)	Reference	Reference	Reference
**Non-violent convictions**			
1^st^ (80–119)	1.26 (1.24, 1.28)	1.22 (1.20, 1.24)	1.20 (1.18, 1.22)
2^nd^ (120–123)	1.18 (1.16, 1.20)	1.15 (1.13, 1.17)	1.14 (1.12, 1.16)
3^rd^ (124–129)	1.13 (1.11, 1.15)	1.10 (1.08, 1.12)	1.09 (1.07, 1.11)
4^th^ (130–137)	1.09 (1.07, 1.10)	1.07 (1.05, 1.08)	1.06 (1.05, 1.08)
5^th^ (138–160)	Reference	Reference	Reference

Abbreviations: mmHg (millimeter of mercury), HR (hazard ratio)

^a^ Adjusted for age at first conviction, birth year, and SES

^b^ Adjusted for age at first conviction, birth year, SES, physical energy capacity, height, and weight

^c^ Category of comparison

### Sensitivity analyses

Estimates were not affected by restricting the analyses to include only men who had their first criminal conviction before conscription, with outcomes of interest occurring after conscription (S1 and S2 Tables). Our estimates were also not affected by restricting the analyses to include only men for whom both their first conviction and the outcome of interest occurring after conscription (S3 and S4 Tables). Further, the estimates were not affected by using age as the underlying temporal metric (S1–S6 Tables) or when restricting the analyses to include only non-violent convictions (S7–S9 Tables).

The estimates were also not affected by using the crime date, instead of the date of conviction, when applicable (S10 and S11 Tables), nor were they affected by restricting the sample only to men with both valid RHR and SBP data (S12 Table).

## Discussion

In a sample of 407,533 convicted male conscripts in Sweden born between 1958 and 1990, we found that lower RHR and lower SBP at age 18 were associated with an increased risk of reoffending. These results replicate findings from previous work conducted on risk factors for criminal behavior [5, 9], and extend these findings in important ways. Specifically, our findings replicate prior work demonstrating that individual differences in autonomic arousal, measured as RHR and SBP, are associated with criminal behavior [5, 9]. However, earlier work has not considered the prior criminal histories of study participants. Our study focused on men with a history of criminal convictions and showed that these indicators of autonomic arousal predicted subsequent reoffending. This finding is important because it provides evidence that variations in autonomic arousal are associated not only with the likelihood of offending [5, 9], but also with the persistence and repetitiveness of offending.

Our findings suggest that low RHR and low SBP should be further investigated and considered as potential predictors to be included in risk assessment protocols. All estimates in the present study remained statistically significant, with some in fact strengthened, after adjusting for pertinent covariates, indicating that RHR and SBP contribute uniquely to prediction of reoffending. Individuals with low RHR who engage in criminal behavior may not benefit from traditional treatment programs to the extent they are, according to theoretical accounts based on low fear [4] and stimulation-seeking propensities [4, 16], less able to learn from their experiences. In particular, low fear of punishment may reduce the effectiveness of conditioning. As evidence for this, youth with disruptive behavior disorders have been found to profit less from behavioral treatment if they also exhibit low RHR [30]. From this perspective, individuals who engage in criminal behavior who also have low RHR may require tailored interventions targeting their under-aroused autonomic nervous system to prevent them from reoffending. Low RHR and low SBP may have limited importance in themselves as predictors of reoffending, but together with more established predictors (e.g., history of criminal behavior, employment, psychiatric disorder) [2], they could add importantly to the identification of individuals at high risk for reoffending who could be prioritized for intervention programs. We encourage future research aimed at incorporating autonomic arousal measures into models for predicting reoffending.

It is well-known that criminal behavior runs in families [23], and that measures of autonomic arousal are heritable [31]. In addition, findings from recent research provide evidence that the association between low RHR and criminal behavior is substantially attributable to genetic influences [9]. This evidence suggests that autonomic arousal as indexed by low RHR and SBP levels may be one of the biological factors underlying transmission of criminal behavior from one generation to the next [8]. Taken together, our findings provide further impetus for considering autonomic arousal variations in etiologic models of reoffending.

The results from the present study are novel, as only one previous small-scale study has tested for an association between low RHR and reoffending [10]. In contrast to our results, this study did not find an association between low RHR and reoffending, potentially due to lack of statistical power. However, this study did find that weak heart rate reactivity and an elevated heart rate variability within a stressor task predicted a higher likelihood of reoffending during follow up. The current study is therefore the first to demonstrate an increased risk of reoffending in a large-scale population-based sample of men with prior conviction histories. Our finding of an increased risk for reoffending among previously convicted men with low SBP is also novel, as no study has examined this association before. Future research should replicate these findings in different settings and populations.

The findings of the present study should be considered in the light of some limitations. RHR and SBP were measured concurrently using an arm-cuff monitor, after subjects had rested for 5 to 10 minutes [21]. This procedure differs from the conventional laboratory method of measuring cardiac activity from skin-surface electrodes attached to the limbs or torso, which may yield cleaner data [32]. However, use of an arm-cuff is the most prevalent method for obtaining these measures clinically, and is standard practice in clinical settings in Sweden [21]. Further, no other information pertaining to the collection of RHR and SBP data was available in the Swedish Military Conscription Register. Therefore, factors such as time of recording and room temperature that may influence cardiovascular measurements could not be controlled for. It also warrants mention that valid RHR data were missing for 43% of the overall registry sample. However, sensitivity analysis including only men with valid RHR and SBP data yielded comparable estimates. This is in line with a prior study examining low RHR in relation to criminal behavior, which showed that excluding the portion of the sample who lacked RHR data did not affect the observed associations [5].

A further limitation is that our study included only men, so it remains to be seen whether our findings generalize to women. Although previous research has shown that low autonomic arousal is associated with criminal behavior for both men and women [7], we cannot draw the same conclusion with respect to reoffending. It is also important to bear in mind that our study focused on men with official convictions only, who may not be representative of all men who have engaged in criminal acts within Sweden.

In conclusion, our findings demonstrate evidence for associations for two distinct indices of autonomic arousal, RHR and SBP, with risk of reoffending, even after adjusting for possible extraneous confounds. These findings indicate that low RHR and low SBP should be further examined as predictors of reoffending, as they may help to improve identification of individuals at risk for repeated criminal justice involvement. Further evidence for the predictive value of autonomic arousal measures would support their inclusion in risk assessment protocols, as a basis for targeting case management and intervention efforts.

## Supporting information

S1 Table Fully adjusted cox proportional hazard regression models for RHR and reoffending as any conviction, violent convictions and non-violent convictions with the first conviction before conscription and reoffending after conscription. (DOCX)

S2 Table Fully adjusted cox proportional hazard regression models for SBP and reoffending as any conviction, violent convictions and non-violent convictions with the first conviction before conscription and reoffending after conscription. (DOCX)

S3 Table Fully adjusted cox proportional hazard regression models for RHR and reoffending as any conviction, violent convictions and non-violent convictions with the first conviction and reoffending after conscription. (DOCX)

S4 Table Fully adjusted cox proportional hazard regression models for SBP and reoffending as any conviction, violent convictions and non-violent convictions with the first conviction and reoffending after conscription. (DOCX)

S5 Table Fully adjusted cox proportional hazard regression models for RHR and reoffending as any conviction, violent convictions and non-violent convictions with first conviction and reoffending at any point in time and age as the underlying temporal metric. (DOCX)

S6 Table Fully adjusted cox proportional hazard regression models for SBP and reoffending as conviction, violent convictions and non-violent convictions with first conviction and reoffending at any point in time and age as the underlying temporal metric. (DOCX)

S7 Table Fully adjusted cox proportional hazard regression models for RHR and SBP with reoffending as non-violent convictions with first conviction and reoffending at any point in time among men with no violent convictions. (DOCX)

S8 Table Fully adjusted cox proportional hazard regression models for RHR and SBP with reoffending as non-violent convictions with first conviction before conscription and reoffending after conscription among men with no violent convictions. (DOCX)

S9 Table Fully adjusted cox proportional hazard regression models for RHR and SBP with reoffending as non-violent convictions with first conviction and reoffending after conscription among men with no violent convictions. (DOCX)

S10 Table Fully adjusted cox proportional hazard regression models for RHR and reoffending as any conviction, violent convictions, and non-violent convictions with first conviction and reoffending at any point in time using date of crime when applicable. (DOCX)

S11 Table Fully adjusted cox proportional hazard regression models for SBP and reoffending as any conviction, violent convictions, and non-violent convictions with first conviction and reoffending at any point in time using date of crime when applicable. (DOCX)

S12 Table Fully adjusted cox proportional hazard regression models for RHR and SBP with reoffending as any conviction, violent convictions and non-violent convictions with the first conviction and reoffending at any point in time among men with valid RHR and SBP data. (DOCX)

S1 Fig Kaplan Meier survival curves for reoffending as any conviction by quintiles of RHR. (TIF)

S2 Fig Kaplan Meier survival curves for reoffending as any conviction by quintiles of SBP. (TIF)
