# Supplementary material for: Low autonomic arousal as a risk factor for reoffending: A population-based study
Source: PLoS One. 2021 Aug 20;16(8):e0256250. doi: 10.1371/journal.pone.0256250 (PMC8378731; doi:10.1371/journal.pone.0256250)
Supplement: S2 Table — (DOCX) [file pone.0256250.s002.docx]

**S2 Table. Fully Adjusted Cox Proportional Hazard Regression Models for SBP and Reoffending as Any Conviction, Violent Convictions and Non-Violent Convictions with the First Conviction before Conscription and Reoffending after Conscription**

|  | **Hazard Ratio (95% CI)** |  | **Hazard Ratio (95% CI)** |
| --- | --- | --- | --- |
| **Quintiles for SBP in mmHg** | **Fully adjusted model ^ac^** | **Quintiles for SBP in mmHg** | **Fully adjusted model ^bc^** |
| **All convictions** |  | **All convictions** |  |
| 1^st^ (80-119) | 1.18 (1.16, 1.21) | 1^st^ (80-119) | 1.18 (1.15, 1.21) |
| 2^nd^ (120-123) | 1.14 (1.11, 1.17) | 2^nd^ (120-123) | 1.13 (1.10, 1.16) |
| 3^rd^ (124-129) | 1.08 (1.06, 1.11) | 3^rd^ (124-129) | 1.08 (1.05, 1.11) |
| 4^th^ (130-137) | 1.07 (1.04, 1.09) | 4^th^ (130-137) | 1.06 (1.04, 1.09) |
| 5^th^ (138-160) | Reference | 5^th^ (138-160) | Reference |
| **Violent convictions** |  | **Violent convictions** |  |
| 1^st^ (80-119) | 1.17 (1.12, 1.21) | 1^st^ (80-119) | 1.16 (1.11, 1.21) |
| 2^nd^ (120-123) | 1.12 (1.07, 1.17) | 2^nd^ (120-123) | 1.12 (1.07, 1.17) |
| 3^rd^ (124-129) | 1.08 (1.04, 1.13) | 3^rd^ (124-129) | 1.08 (1.03, 1.13) |
| 4^th^ (130-137) | 1.08 (1.04, 1.13) | 4^th^ (130-137) | 1.08 (1.04, 1.13) |
| 5^th^ (138-160) | Reference | 5^th^ (138-160) | Reference |
| **Non-violent convictions** |  | **Non-violent convictions** |  |
| 1^st^ (80-119) | 1.19 (1.16, 1.22) | 1^st^ (80-119) | 1.18 (1.15, 1.21) |
| 2^nd^ (120-123) | 1.14 (1.11, 1.17) | 2^nd^ (120-123) | 1.13 (1.10, 1.16) |
| 3^rd^ (124-129) | 1.09 (1.06, 1.11) | 3^rd^ (124-129) | 1.08 (1.06, 1.11) |
| 4^th^ (130-137) | 1.06 (1.04, 1.09) | 4^th^ (130-137) | 1.06 (1.04, 1.09) |
| 5^th^ (138-160) | Reference | 5^th^ (138-160) | Reference |

Abbreviations: SBP (systolic blood pressure), mmHg (millimeter of mercury)

^a^Time since conscription as the underlying time scale

^b^Age as the underlying time scale

^c^Adjusted for birth year, SES, physical capacity, height, and weight
