# Supplementary material for: Low autonomic arousal as a risk factor for reoffending: A population-based study
Source: PLoS One. 2021 Aug 20;16(8):e0256250. doi: 10.1371/journal.pone.0256250 (PMC8378731; doi:10.1371/journal.pone.0256250)
Supplement: S3 Table — (DOCX) [file pone.0256250.s003.docx]

**S3 Table. Fully Adjusted Cox Proportional Hazard Regression Models for RHR and Reoffending as Any Conviction, Violent Convictions and Non-Violent Convictions with the First Conviction and Reoffending after Conscription**

|  | **Hazard Ratio (95% CI)** |  | **Hazard Ratio (95% CI)** |
| --- | --- | --- | --- |
| **Quintiles for RHR in bpm** | **Fully adjusted model^ac^** | **Quintiles for RHR in bpm** | **Fully adjusted model^bd^** |
| **All convictions** |  | **All convictions** |  |
| 1^st^ (35-60) | 1.15 (1.12, 1.19) | 1^st^ (35-60) | 1.15 (1.11, 1.19) |
| 2^nd^ (61-67) | 1.11 (1.08, 1.15) | 2^nd^ (61-67) | 1.11 (1.08, 1.15) |
| 3^rd^ (68-73) | 1.09 (1.06, 1.12) | 3^rd^ (68-73) | 1.08 (1.05, 1.12) |
| 4^th^ (74-81) | 1.07 (1.03, 1.10) | 4^th^ (74-81) | 1.06 (1.03, 1.09) |
| 5^th^ (82-145) | Reference | 5^th^ (82-145) | Reference |
| **Violent convictions** |  | **Violent convictions** |  |
| 1^st^ (35-60) | 1.21 (1.09, 1.34) | 1^st^ (35-60) | 1.21 (1.09, 1.34) |
| 2^nd^ (61-67) | 1.18 (1.07, 1.30) | 2^nd^ (61-67) | 1.18 (1.07, 1.31) |
| 3^rd^ (68-73) | 1.00 (0.90, 1.11) | 3^rd^ (68-73) | 1.00 (0.90, 1.11) |
| 4^th^ (74-81) | 1.02 (0.92, 1.12) | 4^th^ (74-81) | 1.02 (0.92, 1.13) |
| 5^th^ (82-145) | Reference | 5^th^ (82-145) | Reference |
| **Non-violent convictions** |  | **Non-violent convictions** |  |
| 1^st^ (35-60) | 1.14 (1.10, 1.18) | 1^st^ (35-60) | 1.13 (1.09, 1.17) |
| 2^nd^ (61-67) | 1.11 (1.08, 1.15) | 2^nd^ (61-67) | 1.11 (1.07, 1.14) |
| 3^rd^ (68-73) | 1.09 (1.06, 1.13) | 3^rd^ (68-73) | 1.09 (1.05, 1.12) |
| 4^th^ (74-81) | 1.06 (1.03, 1.09) | 4^th^ (74-81) | 1.05 (1.02, 1.09) |
| 5^th^ (82-145) | Reference | 5^th^ (82-145) | Reference |

Abbreviations: RHR (resting heart rate), bpm (beats per minute)

^a^Time since first crime as the underlying time scale

^b^Age as the underlying time scale

^c^Adjusted for age at first crime, birth year, SES, physical capacity, height, and weight

^d^Adjusted for birth year, SES, physical capacity, height, and weight
