# Supplementary material for: Low autonomic arousal as a risk factor for reoffending: A population-based study
Source: PLoS One. 2021 Aug 20;16(8):e0256250. doi: 10.1371/journal.pone.0256250 (PMC8378731; doi:10.1371/journal.pone.0256250)
Supplement: S5 Table — (DOCX) [file pone.0256250.s005.docx]

**S5 Table. Fully Adjusted Cox Proportional Hazard Regression Models for RHR and Reoffending as Any Conviction, Violent Convictions and Non-Violent Convictions with First Conviction and Reoffending at Any Point in Time and Age as the Underlying Temporal Metric**

|  | **Hazard Ratio (95% CI)** |
| --- | --- |
| **Quintiles for RHR in bpm** | **Fully adjusted model^a^** |
| **All convictions** |  |
| 1^st^ (35-60) | 1.16 (1.14, 1.19) |
| 2^nd^ (61-67) | 1.11 (1.09, 1.14) |
| 3^rd^ (68-73) | 1.09 (1.07, 1.12) |
| 4^th^ (74-81) | 1.05 (1.03, 1.08) |
| 5^th^ (82-145) | Reference |
| **Violent convictions** |  |
| 1^st^ (35-60) | 1.24 (1.18, 1.31) |
| 2^nd^ (61-67) | 1.20 (1.14, 1.26) |
| 3^rd^ (68-73) | 1.09 (1.04, 1.15) |
| 4^th^ (74-81) | 1.05 (1.00, 1.10) |
| 5^th^ (82-145) | Reference |
| **Non-violent convictions** |  |
| 1^st^ (35-60) | 1.16 (1.13, 1.19) |
| 2^nd^ (61-67) | 1.11 (1.08, 1.14) |
| 3^rd^ (68-73) | 1.09 (1.07, 1.12) |
| 4^th^ (74-81) | 1.05 (1.03, 1.08) |
| 5^th^ (82-145) | Reference |

Abbreviations: RHR (resting heart rate), bpm (beats per minute)

^a^Adjusted for birth year, SES, physical capacity, height, and weight
