# Supplementary material for: Low autonomic arousal as a risk factor for reoffending: A population-based study
Source: PLoS One. 2021 Aug 20;16(8):e0256250. doi: 10.1371/journal.pone.0256250 (PMC8378731; doi:10.1371/journal.pone.0256250)
Supplement: S7 Table — (DOCX) [file pone.0256250.s007.docx]

**S7 Table. Fully Adjusted Cox Proportional Hazard Regression Models for RHR and SBP with Reoffending as Non-Violent Convictions with First Conviction and Reoffending at Any Point in Time among Men with No Violent Convictions**

|  | **Hazard Ratio (95% CI)** |  | **Hazard Ratio (95% CI)** |
| --- | --- | --- | --- |
|  | **Fully adjusted model^ac^** |  | **Fully adjusted model^bd^** |
| **Quintiles for RHR in bpm** |  | **Quintiles for RHR in bpm** |  |
| 1^st^ (35-60) | 1.09 (1.06, 1.12) | 1^st^ (35-60) | 1.07 (1.04, 1.10) |
| 2^nd^ (61-67) | 1.06 (1.03, 1.09) | 2^nd^ (61-67) | 1.05 (1.02, 1.08) |
| 3^rd^ (68-73) | 1.07 (1.04, 1.09) | 3^rd^ (68-73) | 1.05 (1.03, 1.08) |
| 4^th^ (74-81) | 1.05 (1.02, 1.08) | 4^th^ (74-81) | 1.04 (1.01, 1.07) |
| 5^th^ (82-145) | Reference | 5^th^ (82-145) | Reference |
| **Quintiles for SBP in mmHg** |  | **Quintiles for SBP in mmHg** |  |
| 1^st^ (80-119) | 1.16 (1.13, 1.18) | 1^st^ (80-119) | 1.13 (1.11, 1.16) |
| 2^nd^ (120-123) | 1.12 (1.09, 1.14) | 2^nd^ (120-123) | 1.10 (1.08, 1.13) |
| 3^rd^ (124-129) | 1.07 (1.05, 1.09) | 3^rd^ (124-129) | 1.06 (1.04, 1.08) |
| 4^th^ (130-137) | 1.04 (1.02, 1.06) | 4^th^ (130-137) | 1.03 (1.01, 1.05) |
| 5^th^ (138-160) | Reference | 5^th^ (138-160) | Reference |

Abbreviations: RHR (resting heart rate), bpm (beats per minute), SBP (systolic blood pressure), mmHg (millimeter of mercury)

^a^Time since first crime as the underlying time scale

^b^Age as the underlying time scale

^c^Adjusted for age at first crime, birth year, SES, physical capacity, height, and weight

^d^Adjusted for birth year, SES, physical capacity, height, and weight
