# Supplementary material for: Low autonomic arousal as a risk factor for reoffending: A population-based study
Source: PLoS One. 2021 Aug 20;16(8):e0256250. doi: 10.1371/journal.pone.0256250 (PMC8378731; doi:10.1371/journal.pone.0256250)
Supplement: S12 Table — (DOCX) [file pone.0256250.s012.docx]

**S12 Table. Fully Adjusted Cox Proportional Hazard Regression Models for RHR and SBP with Reoffending as Any Conviction, Violent Convictions and Non-Violent Convictions with the First Conviction and Reoffending at Any Point in Time Among Men with Valid RHR and SBP Data**

|  | **Hazard Ratio (95% CI)** |  | **Hazard Ratio (95% CI)** |
| --- | --- | --- | --- |
| **Quintiles for RHR in**  **bpm** | **Fully adjusted model^ab^** | **Quintiles for SBP in mmHg** | **Fully adjusted model^ab^** |
| **All convictions** |  | **All convictions** |  |
| 1^st^ (35-60) | 1.17 (1.14, 1.19) | 1^st^ (80-119) | 1.18 (1.15, 1.20) |
| 2^nd^ (61-67) | 1.12 (1.09, 1.14) | 2^nd^ (120-123) | 1.12 (1.10, 1.15) |
| 3^rd^ (68-73) | 1.09 (1.07, 1.12) | 3^rd^ (124-129) | 1.09 (1.06, 1.11) |
| 4^th^ (74-81) | 1.06 (1.04, 1.08) | 4^th^ (130-137) | 1.05 (1.03, 1.08) |
| 5^th^ (82-145) | Reference | 5^th^ (138-160) | Reference |
| **Violent convictions** |  | **Violent convictions** |  |
| 1^st^ (35-60) | 1.23 (1.17, 1.29) | 1^st^ (80-119) | 1.14 (1.08, 1.20) |
| 2^nd^ (61-67) | 1.18 (1.13, 1.24) | 2^nd^ (120-123) | 1.08 (1.02, 1.14) |
| 3^rd^ (68-73) | 1.09 (1.03, 1.14) | 3^rd^ (124-129) | 1.05 (1.00, 1.11) |
| 4^th^ (74-81) | 1.04 (0.99, 1.09) | 4^th^ (130-137) | 1.06 (1.01, 1.11) |
| 5^th^ (82-145) | Reference | 5^th^ (138-160) | Reference |
| **Non-violent convictions** |  | **Non-violent convictions** |  |
| 1^st^ (35-60) | 1.16 (1.13, 1.19) | 1^st^ (80-119) | 1.18 (1.15, 1.21) |
| 2^nd^ (61-67) | 1.11 (1.09, 1.14) | 2^nd^ (120-123) | 1.13 (1.10, 1.16) |
| 3^rd^ (68-73) | 1.10 (1.07, 1.12) | 3^rd^ (124-129) | 1.09 (1.06, 1.11) |
| 4^th^ (74-81) | 1.05 (1.03, 1.08) | 4^th^ (130-137) | 1.05 (1.03, 1.08) |
| 5^th^ (82-145) | Reference | 5^th^ (138-160) | Reference |

Abbreviations: RHR (resting heart rate), bpm (beats per minute), SBP (systolic blood pressure), mmHg (millimeter of mercury)

^a^Time since first crime as the underlying temporal metric

^b^Adjusted for age at first crime, birth year, SES, physical capacity, height, and weight
