# Supplementary figures and images for: Low autonomic arousal as a risk factor for reoffending: A population-based study
Source: PLoS One. 2021 Aug 20;16(8):e0256250. doi: 10.1371/journal.pone.0256250 (PMC8378731; doi:10.1371/journal.pone.0256250)

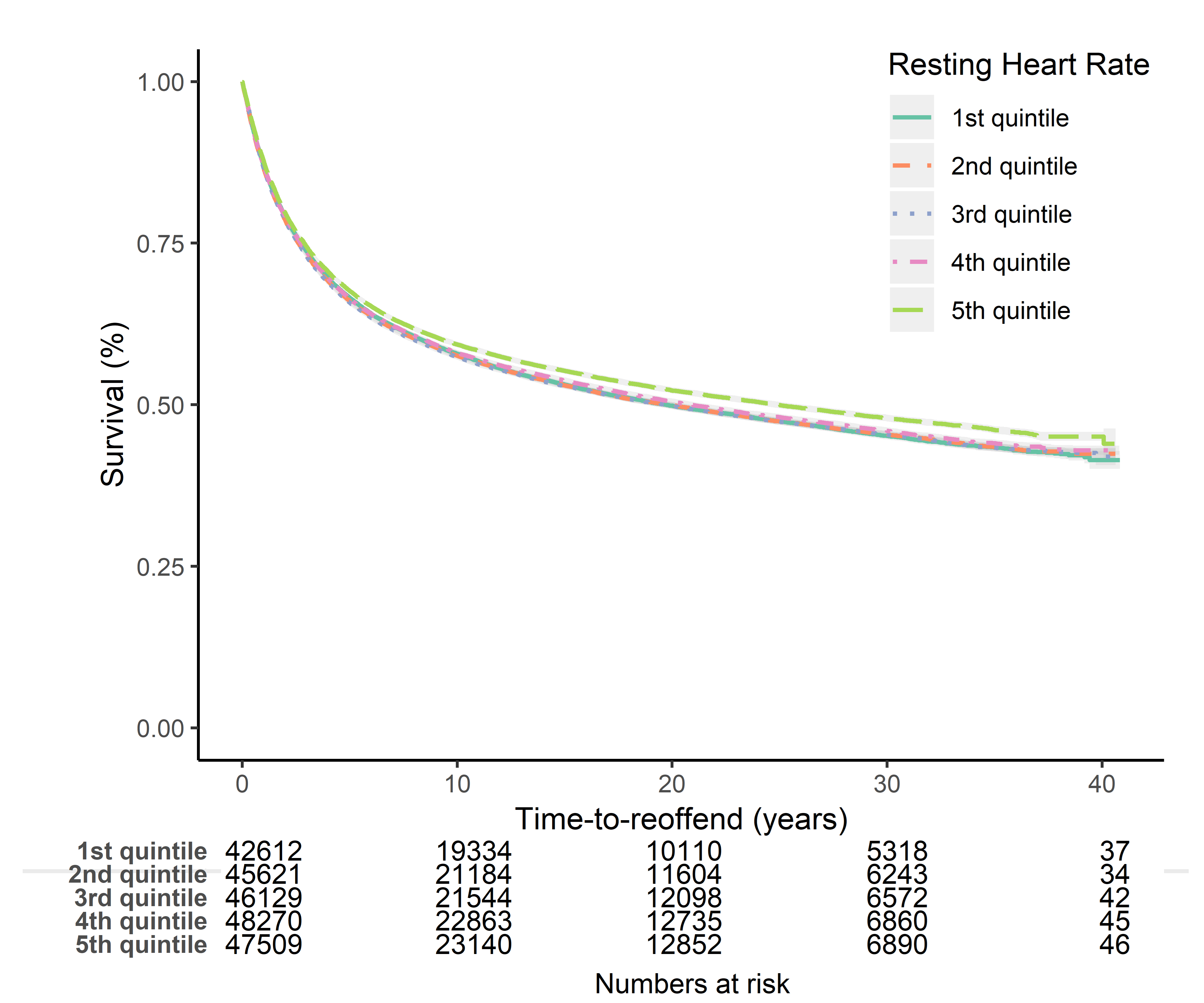

Supplement: S1 Fig — (TIF) [file pone.0256250.s013.tif]

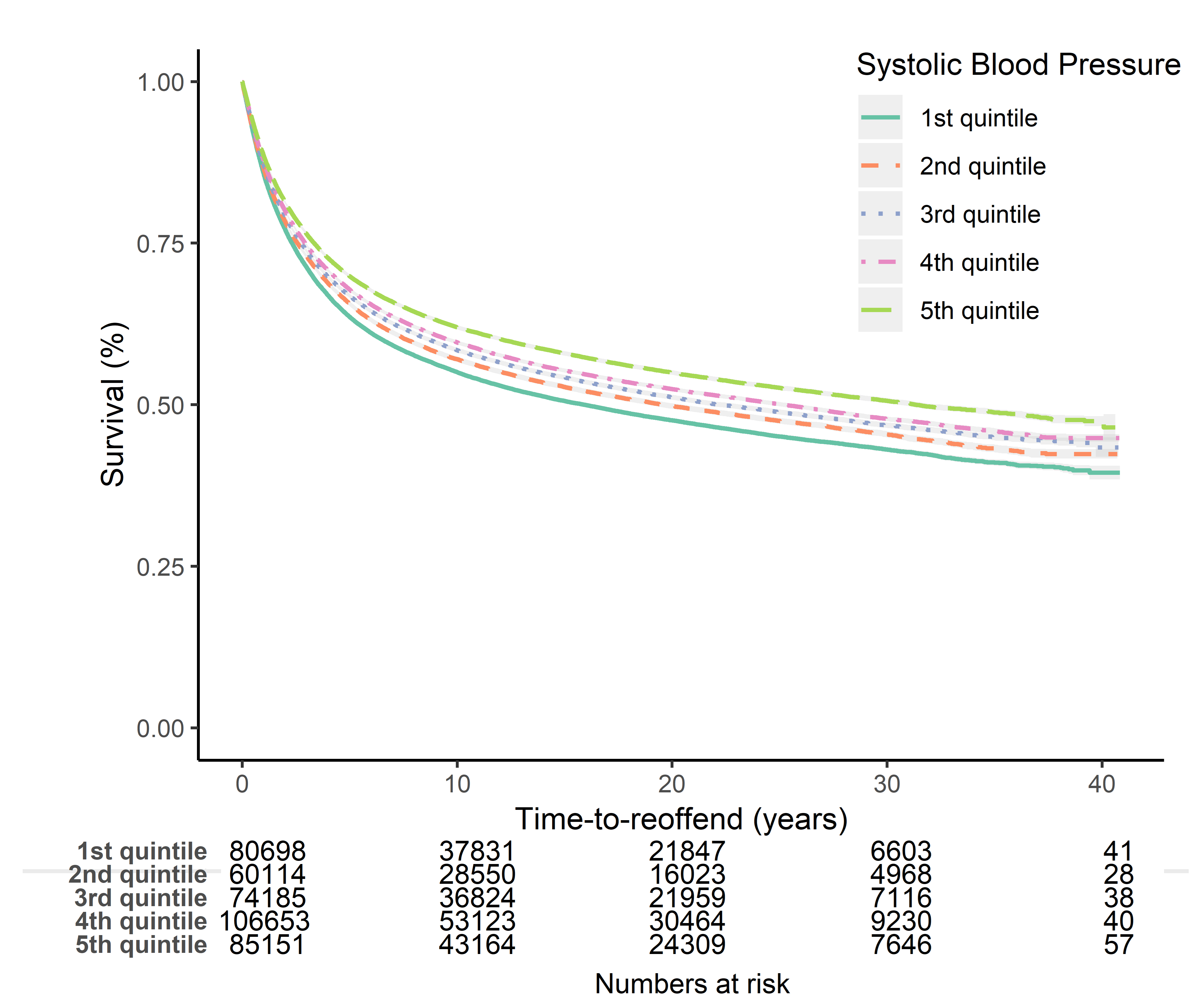

Supplement: S2 Fig — (TIF) [file pone.0256250.s014.tif]
